# Non-Steroidal Drug Interferences in a Quantitative Multisteroid LC-MS/MS Assay

**DOI:** 10.3390/cells12020329

**Published:** 2023-01-15

**Authors:** Valentin Braun, Hermann Stuppner, Christoph Seger

**Affiliations:** 1 Institute of Pharmacy/Pharmacognosy, CCB—Centrum of Chemistry and Biomedicine, University of Innsbruck, Innrain 80-82, A-6020 Innsbruck, Austria; 2 Dr. Risch Ostschweiz AG, Lagerstrasse 30, 9470 Buchs, Switzerland

**Keywords:** steroid, LC-MS/MS, 17-hydroxyprogesterone, aldosterone, paroxetine, α-hydroxytriazolam, triazolam, interference, drug, non-steroidal

## Abstract

Screening for possible interferences from steroidal compounds other than the target analytes (endogenous or exogenous) is well established in LC-MS/MS assay development for steroid quantification in a routine clinical setting. However, interferences from non-steroidal substances have, hitherto, not been explored. After screening more than 150 pharmaceuticals and their metabolites by analyzing commercial quality control samples from TDM analysis kits (Recipe, Chromsystems) with a multisteroid LC-MS/MS assay (protein precipitation followed by HybridSPE filtration, biphenyl column, methanol–water gradient with NH4F additive), we can report the finding of two newly discovered potential interferences from non-steroidal drugs. Antidepressant paroxetine (PX) was identified as an interference to 17-hydroxyprogesterone (17P), and α-hydroxytriazolam (α-OH-TZM)—a major metabolite of benzodiazepine triazolam (TZM)—was identified as an interference to aldosterone (ALDO). Despite different elemental and structural compositions and nominal masses, the M+1 isotopologues of PX and α-OH-TZM produced overlapping signals in ion traces monitored for the respective analytes (m/z 331 → 109/97 and 361→315/343, respectively). PX and TZM are frequently prescribed drugs, and their therapeutic ranges are far exceeding the reference ranges of 17P or ALDO (µmol vs nmol); therefore, these interferences should be considered clinically relevant. Striving for faster multi-analyte methods with high sample turnover, especially in the field of steroid quantification, can limit assay selectivity and specificity. Therefore, supported by the findings of this study, screening for potential interferences in multi-analyte LC-MS/MS method development should not cover only substances of the same class but also include a set of common drugs.

## 1. Introduction

Mass spectrometric analysis, preferably the detection of small organic molecules after chromatographic separation by means of two-stage mass spectrometric detection (HPLC-MS/MS), has become well established in clinical routines in recent years [1]. Usually, spectrometers are used, which have mass filters based on quadrupoles. In “selected reaction monitoring” (SRM), the dissociation of the target molecules in the gas phase is used to ensure high analytical selectivity, which should lead to specific substance detection. In SRM experiments, the first quadrupole is set such that only ions of the molecular mass of the target molecule, which are formed in the ion source, can pass. Between the two quadrupoles, a kinetic fragmentation of the target molecule is provoked in a reaction cell. In each experiment, one of the reaction products (a “fragment ion”), which is as analyte-specific as possible, is selected in the second quadrupole.

Typically, more than one SRM experiment is performed to allow for the reciprocal confirmation of the ion yields found. One of the ion traces of these SRM experiments is usually called the quantifier (QN). Via this ion trace, the quantitative analysis result is worked out after validation. One or more additional ion traces are used to confirm the quantitative result. They are, therefore, referred to as qualifiers (QLs). The ion yield ratio QN/QL is often referred to as the “branching ratio” [2] and should be constant under the given experimental conditions. Deviations from this ratio, therefore, indicate the presence of interferences, as do deviations from the ideal peak shape in one of the ion traces.

The operational parameters suitable for the optimal detection of a specific target molecule are determined in preliminary experiments, which are carried out with pure substances of the target molecules. The final measurement instructions for the mass spectrometer consist of ionization conditions for the ion source, filter parameters for the quadrupoles and kinetic parameters for the gas-phase reaction. In a series of experimental tests, the selectivity of the established SRM mass transfer is tested in the design phase of assay development before it is verified in method validation.

It is well known that mass spectrometric separation is not possible for molecules of the same molecular formula (e.g., enantiomers, diastereomers, positional isomers, etc.) without additional selectivity, e.g., chromatographic pre-separation. Often, but not always, additional selectivity in the mass spectrometric domain, e.g., gas-phase reaction selectivity, can be used to overcome this limitation. If a low-resolution mass spectrometer, such as a quadrupole-based tandem MS instrument, is used for detection, the number of potentially interfering substances increases from those with the same molecular formula to all molecules with the same nominal mass. Additionally, the isotopologues—M+1 and M+2—can contribute to interferences, as observed, for example, in the case of cortisol/cortisone/prednisolone/prednisone [3]. This problem can, in principle, be pushed back using high-resolution mass selectors [4], but to date, these have not become widely accepted in clinical routines aside from toxicology.

A fundamental problem of method establishment, completely independent of whether this takes place in an industrial setting or in a singular laboratory, is that, fundamentally, not all conceivable interferences can be tested during method development. This requires good monitoring of the method during its life cycle. Deviating results due to interferences (=individual, sample-specific bias contribution) must be detectable and analyzable. As stated above, in routine diagnostics, the recording of more than one fragment ion (QN/QL) has proven to be useful. If the branching ratio (covering the analyte SRM ion traces) or quantitative results (this includes interferences in the internal standard SRM ion traces) derived from QN/QL recording differ from each other more than the validated threshold (e.g., six standard deviations), the result must be classified as not valid, and a root cause analysis must be started.

Especially in the application field of endogenous steroidal molecules, it is known that due to the multitude of biochemical metabolization pathways and the simultaneously very limited structural diversity (definition of the molecule class “steroids” to terpenoid backbones with 18 to 21 carbon atoms and exclusively oxygen substitutions), interferences by isobaric molecules and their derived metabolites are to be expected [5]. For this reason, in method establishment, common and known endogenous steroidal analytes and exogenous steroidal drugs—if available—are usually tested for their interference potential. In a multi-analyte method recently published by our group, we could show that practically every steroidal substance tested led to a detectable signal and that corresponding precautions had to be taken in the method design to ensure that analyte detection was possible without steroid/steroid detection interference [6].

When testing for the possibility of non-steroidal interferences, a distinction must also be made between endogenous and exogenous substances (xenobiotics). Endogenous interferences can usually be covered by analyzing a set of authentic matrix samples. A suitable variety of materials from healthy and diseased persons, which covers the application range of the assay, must be investigated in method establishment, either as single samples or as pool samples. Compared to controlled clinical studies, a much more detailed investigation must be considered in the context of method development for routine clinical applications, as the diversity of human samples usually exceeds the uniformity of healthy study participants.

Exogenous interferences are more difficult to screen. It is of course possible to spike human samples with the drugs and substances that are expected to be used in clinical routine. This allows a large variety of substance classes to be covered, but in principle, it does not cover the metabolites of these substances that are expected to be in circulation. It is well known in laboratory diagnostics that such substances can lead to a variety of clinically relevant interferences. For example, the signal cancellation of enzymatic creatinine measurements by metamizole [7], the false positive amphetamine results in patients on trazodone therapy triggered by the trazodone metabolite mCPP [8], or the false high LC-MS/MS results for mycophenolic acid (MPA) when MPA and the metabolite MPAG, breaking down to MPA in the ion source, are not separated chromatographically [9].

As part of the method development of the above-mentioned assay [6], we decided to test more than 150 drugs and their major metabolites, which are known to the clinical laboratory in the context of therapeutic drug monitoring (TDM), for their potential interaction with the target analytes. The materials available to us for this purpose were IVD-CE-certified control materials from two manufacturers, which show widespread application in routine diagnostics. These materials have a native steroid background, and the working hypothesis was that no interference should occur because there were no isobaric drugs in the set and because the molecular structures of the analytes do not correspond to those of the target molecules. In the present study, we show that interferences can nevertheless occur under these experimental conditions, suggesting that the selectivity of the SRM experiment in the analysis of steroidal molecules has distinct experimental limitations.

## 2. Materials and Methods

### 2.1. Materials

Quality control materials and internal standard solutions of LC-MS/MS TDM kits for antidepressants, neuroleptics, antiepileptics, tricyclic antidepressants and antiarrhythmics were purchased from Chromsystems (Gräfeling, Germany), and kits for antidepressants and benzodiazepines were purchased from Recipe (Munich, Germany). Paroxetine (PX) (as hydrochloride hemihydrate) was purchased as a calibrated solution (1 mg/mL base in methanol) from Lipomed (Arlesheim, Switzerland), and α-hydroxytriazolam (α-OH-TZM) was bought as a reference solution (1 mg/mL in methanol) from Cerilliant (Merck/Sigma Aldrich, Buchs SG, Switzerland).

### 2.2. LC-MS/MS Method

All experiments were performed with a method that is capable of quantifying fifteen steroids in a single run and is described in detail elsewhere [6]. In short, the assay characteristics are here described for aldosterone (ALDO) and 17-hydroxyprogesterone (17P)—the analytes covered by this work. Prior to analysis, 100 µL of a calibrator, QC or serum sample were spiked with 20 µL of an internal standard solution (Chromsystems) and then subjected to protein precipitation by a methanolic zinc sulphate solution followed by phospholipid removal by the means of HybridSPE (Merck/Sigma Aldrich). A 40 µL aliquot of the purified sample was used for analysis with the LC-MS/MS system (Sciex API6500+ triple quad mass spectrometer hyphenated to an Agilent 1290 Infinity II UHPLC). Chromatographic separation was performed on a biphenyl stationary phase (Restek Raptor Biphenyl, 100 mm × 2.1 mm, 2.7 µm, BGB Analytik, Boeckten, Switzerland) by gradient elution (water/methanol with 0.2 mM NH4F as additive), resulting in retention times of 2.8 min (ALDO) and 3.8 min (17P). Analyte detection was performed in the positive ESI mode for both analytes, recording two mass transitions each starting from the respective [M+H]^+^ peaks (ALDO: *m*/*z* 361.3 → 315.2/343.2; 17P: *m*/*z* 331.3 → 97.0/109.1).

### 2.3. Screening for and Identification of Interferences

High-level quality control materials of all LC-MS/MS analysis kits used in our laboratory for routine analysis in therapeutic drug monitoring (TDM), containing more than 150 pharmaceuticals and their metabolites at concentrations above the therapeutic range, were screened for potential interferences from non-steroidal substances. Sample preparation was performed as described above. Compounds included in the TDM kits are listed in Table S1. Interference from the tested compounds was suspected if an additional peak or changes in peak shape, signal-to-noise ratio or branching ratio were observed in any of the monitored mass transitions (QN, QL and IS) in comparison to QC or calibrator materials.

### 2.4. Confirmation of Found Interferences

Suspected interferences were further investigated by using reference solutions. Direct injection of diluted reference solutions allowed the recording of product ion spectra, confirming that fragment ions with the same m/z ratio are produced as used for steroid analysis. The identities of found interferences were confirmed by comparing the retention times of the interfering peak-to-peak retention times analyzing reference substances and their respective internal standards recording the mass transitions of affected steroid analytes as well as mass transitions of the interfering compound. Furthermore, for the investigation of the clinical relevance of the interferents, the leftovers of patient samples with known drug content were analyzed with the steroid assay. The patient samples were analyzed using TDM kits described above during routine testing in the laboratory and were de-identified before using leftovers in this experiment.

## 3. Results

As the assay has a sample preparation (protein precipitation followed by phospholipid-removing SPE), which does not enable the separation of salts, acids or bases, it must be expected that common pharmaceuticals are still present in the final sample extract injected into the LC-MS/MS system. Only non-lipophilic drugs and hydrophilic metabolites thereof will elute prior to the steroid congeners, most drugs, however, must be considered as potential sources of interferences [10]. Most pharmaceuticals screened in this experiment (see Table S1) have therapeutic concentration ranges in the high nanomolar or micromolar range, which is much higher than most reference ranges of steroid hormones. Therefore, it can be expected that the M+1, M+2 or M+4 masses of pharmaceuticals (isotopologues) due to naturally occurring isotopes, such as ^13^C, ^15^N, ^37^Cl or ^81^Br, can also produce signals high enough to interfere with the ion transitions of steroid congeners.

Screening the high-level quality control materials of commercial IVD-CE-certified TDM kits from two providers and containing more than 150 single compounds, two interfering substances were identified for two of the fifteen steroids monitored. One co-eluting interfering signal was found in both the QN and QL ion transitions of 17P and one in both the QN and QL transitions of ALDO. The 17P case was found in two IVD kits covering “antidepressant drugs”, and the ALDO case was associated with the “benzodiazepine” kit of one manufacturer. In the sense of a root cause analysis, both interferences were investigated further.

### 3.1. Interference in 17P Analysis

**Figure 1:**
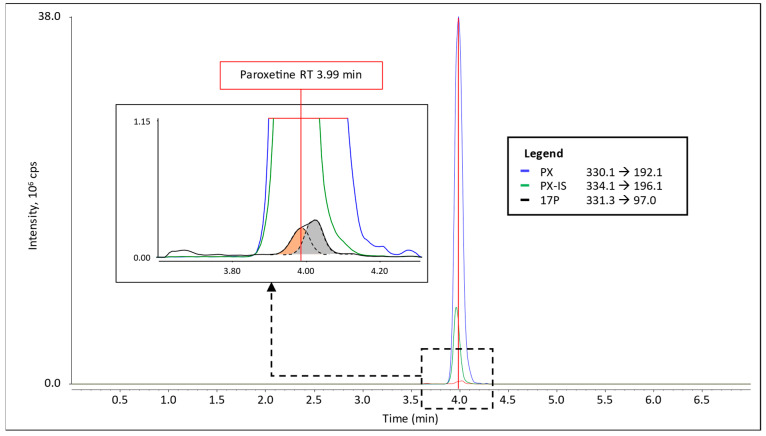
Ion trace chromatograms of a quality control sample of TDM kit “antidepressant panel 1” (Chromsystems) containing PX, PX-IS and 17P. The 17P ion transition is enlarged in the inset box. The 17P peak (grey) has a shoulder originating from co-eluting PX (orange). The retention time of the PX signal in the specific ion transition (330→192) is marked with a red line.

The interference in the 17P transitions was suspected to be an M+1 isotopologue of the antidepressant drug PX as it is included in both antidepressant kits (Chromsystems and Recipe) and the only candidate with a molecular mass similar to 17P (for chemical structures and information, see Scheme S1). Reanalyzing the TDM kit QC samples monitoring the specific ion transitions of PX and of a deuterium-labeled internal standard of PX (PX-IS) did confirm that the co-eluting interference present as a shoulder in the 17P signal had the same retention time as the peak in the specific ion transitions for PX and PX-IS (see Figure 1). Recording Q1 and product ion spectra infusing a pure solution of PX did prove that the same fragment ions, as detected for 17P quantification (97 and 109), can be generated from the M+1 isotopologue of PX (see Figure S1).

**Figure 2:**
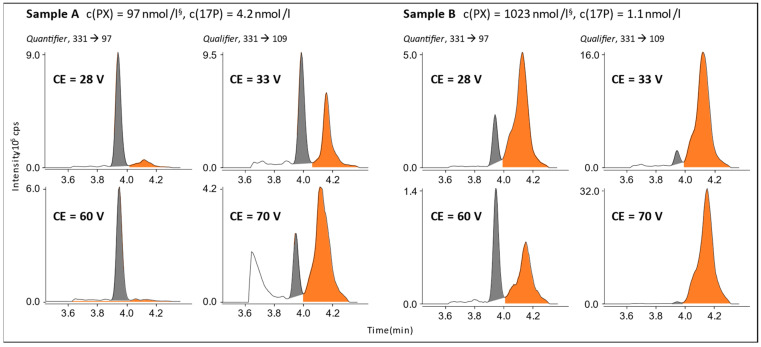
Effect of a raise in collision energy (CE) for the quantifier and qualifier mass transition of 17P on the signal intensities of 17P (grey) and PX (orange) in patients’ samples with known PX concentrations. ^§^ PX concentration was measured during routine TDM testing using an IVD-CE-certified LC-MS/MS assay.

When analyzing patient samples from routine TDM testing with a known PX concentration, an additional signal was found in both 17P ion transition chromatograms. However, in this experiment, PX was well separated from the 17P signal with the only difference being that another column (new LOT) was used (see Figure 2).

After injecting solutions of PX and 17P in neat solvent using three different column LOTs (internal numbering: #6, #7 and #8), a high column-to-column retention time difference for the PX peak of higher than 1 min was observed. Additionally, the matrix in which PX was injected had an influence on the retention time (see Figure S2). In comparison, RTs for 17P stayed almost the same between columns and matrices with RT differences lower than 0.2 min. Matrix and column-dependent retention time shifts were reported in a previous study for PX [11]. The chromatographic behavior of PX could only be stabilized using buffered mobile phases. Therefore, the authors attributed the RT shifts to the pH-dependent nature of the secondary amine of the piperidine substructure of PX [11].

As demonstrated before for androstenedione, altering the collision energy can have a significant impact on selectivity against co-eluting substances [6]. For the QN transition of 17P, a raise of CE value from optimal 28V to 60V was promising, as 17P and PX did show different CE ramp profiles. The effect of the collision energy on signal intensity differences was studied in patient samples from the laboratory archive, which were analyzed in routine testing for their PX content by an IVD-CE-certified LC-MS/MS assay (Chromsystems or Recipe). As depicted in Figure 2, the 17P/PX peak area ratio did increase, and the PX signal was almost fully suppressed at around 100 nmol/L, but at levels greater than 1 µmol/L, a strong interference signal was still present in the quantifier transition of 17P. The qualifier transition was affected in the opposite way with even higher signal intensities for PX in comparison to 17P.

### 3.2. Interference in ALDO Analysis

**Figure 3:**
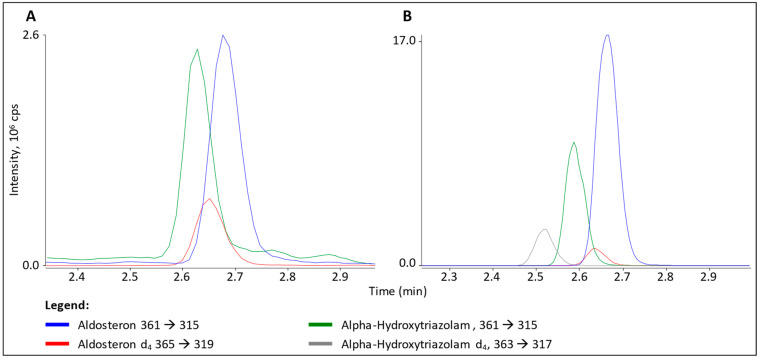
(**A**) α-OH-TZM as observed in ALDO quantifier ion trace (green) screening a QC sample of a benzodiazepine TDM kit (Recipe). ALDO quantifier (blue) and IS (red) ion trace of a high calibrator sample are overlayed for comparison. (**B**) Overlay of ion trace chromatograms from analyzing pure solutions of ALDO (blue), α-OH-TZM (green) and their respective d_4_ labelled internal standards (red and grey).

According to the list of substances included in the benzodiazepines kit, the only candidate for interference in the ALDO mass transitions was α-OH-TZM (C_17_H_12_Cl_2_N_4_O) with an exact mass of 359.2 g/mol and a nominal mass of 358 g/mol (see scheme S1 for more chemical information). Investigating the chemical structure and calculating the isotopic pattern (Figure S3), it is obvious that approximately one-third of all α-OH-TZM molecules present have the same molecular mass as ALDO (360 g/mol). This can be explained by the presence of two chlorine atoms in α-OH-TZM. Due to the natural ^35^Cl-to-^37^Cl isotope ratio of three to one, two chlorine atoms result in an M-to-M+2 ratio of approximately five to three. Injecting pure solutions of α-OH-TZM, ALDO and the respective deuterated internal standards did prove this theory. ALDO produced a signal only about twice as high as the α-OH-TZM signal (see Figure 3B). Although partially separated, the ALDO signal was completely masked in the high-QC sample of the benzodiazepine TDM kit with an α-OH-TZM concentration of 150 nmol/L (see Figure 3A).

## 4. Discussion

### 4.1. Paroxetine

A multitude of LC-MS/MS assays are described for the quantification of 17P in human serum or plasma, assays for routine use in clinical laboratories as well as (candidate) reference methods [12]. To the best of our knowledge, most assays were tested for interferences from several steroidal compounds, but no assay was checked specifically for PX or screened for other common non-steroidal pharmaceuticals.

In our experiments, PX was shown to have a high potential for interfering with the quantification of 17P. It has a high RT variability when using unbuffered mobile phases depending on the sample matrix and column age or LOT. This makes PX an interference of concern not only in the presented assay using a biphenyl stationary phase but also for 17P LC-MS/MS assays, in general, using, for example, the more common C_18_ stationary phases. Even though being only an isotopic M+1 interference, PX produces significant signals in the mass transitions of 17P, as it can reach blood levels with an average C_max_ of 190 nmol/L, which is about a hundred times higher than the reference ranges of 17P [13].

It should also be considered that PX could interfere with other steroid analytes isobaric to 17P that are usually measured using the same mass transitions, e.g., 11-deoxycorticosterone (11-DOC), which was also covered by our steroid assay. However, in this analytical setup, an interference of the 11-DOC signal by PX was not observed. Nevertheless, due to the unstable RT of PX, it could not be excluded either.

Patients regularly tested for their 17P levels, e.g., with diagnosed congenital adrenal hyperplasia (CAH) or polycystic ovary syndrome (PCOS) patients, also have a significantly higher chance of getting diagnosed with depression and, therefore, being treated with antidepressants such as PX [14,15]. A study in Denmark on PCOS patients found 16% to be treated by antidepressant drugs in comparison to 8% in a control group [16]. Another study found that depression is diagnosed four times more often in PCOS patients [15]. There were no data available on the prescriptions of PX in the PCOS or CAH patients’ group. However, PX accounted for around 3% of all antidepressants defined daily doses (DDD) and for 6% of all DDD in the subgroup of selective serotonin reuptake inhibitors (SSRI) in Germany in the year 2019 [17]. Similar statistics are available for the US [18]. According to the prescription database of the Norwegian Health Institute, PX accounts for about 10% of all SSRI prescriptions, and around 0.25% of the total population was treated with PX in the last 5 years [19]. If around 15% of PCOS and CAH patients are treated with antidepressants, and if PX is prescribed in a similar frequency as in the overall population, it can be estimated that a significant share of total PCOS or CAH samples could be affected by PX interference (up to 1%), potentially leading to falsely high or false positive results. This could have clinically significant consequences for the single patient as diagnosis and therapy of PCOS and CAH heavily relies on the biochemical testing of 17P.

If a separation of PX cannot be achieved, the best way to deal with the PX interference—aside from pre-analytical measures—is to monitor the QN/QL ion ratio of 17P. While the ion ratio in unaffected 17P measurements was around 1.0, the ratio dropped to around 0.5 in samples with co-eluting PX. If the assay has a sufficient sensitivity budget, the increase of the CE value could also increase the specificity of 17P measurements against PX and probably also against other potential interferences. A column or column LOT providing a constant baseline separation of 17P and PX was identified and kept on hand for a reanalysis of the affected samples.

### 4.2. α-Hydroxytriazolam

A-OH-TZM is the major metabolite of the benzodiazepine triazolam, also known under the brand name Halcion^®^. Normal plasma levels of the original drug are between 5.5 and 55 nmol/L after a usual oral dose of triazolam of 0.25 mg [20]. The metabolite α-OH-TZM reaches around two-thirds of the plasma levels of its parent drug, with a C_max_ after 1 h and a t_1/2_ of 6 to 8 h [21]. As ALDO concentrations are usually <0.5 nmol/L, quantification would be significantly impaired in patients treated with triazolam. However, according to prescription databases of different countries, triazolam is a rather rarely prescribed drug (at least compared to PX mentioned above), and its use has been decreasing in recent years [17,19]. Therefore, α-OH-TZM is most probably not of high concern as interference in day-to-day routine ALDO analysis in this study’s method or other LC-MS/MS methods. Nevertheless, α-OH-TZM must still be considered as a clinically relevant interference, since ALDO analysis of single patients could be severely affected, especially looking at the potential of TZM for dependence and abuse [22]. Furthermore, to the best of our knowledge, α-OH-TZM was not described as an ALDO interference before, and it is a good example that not only drugs themselves but also their major metabolites should be screened in assay development.

## 5. Conclusions

We presented, hitherto, undescribed interferents in the LC-MS/MS analysis of ALDO and 17P. Interferences from endogenous or exogenous steroidal congeners are quite common and must be expected in a fast multisteroid assay with a sample preparation providing only limited selectivity. However, to the best of our knowledge, no interferences have ever been described from pharmaceuticals of non-steroidal nature, neither in the LC-MS/MS analysis of 17P and ALDO nor of steroids in general.

Therefore, we not only recommend checking every existing or future 17P or ALDO LC-MS/MS assay if PX or α-OH-TZM are potential interferences but also screening for non-steroidal interferences generally during LC-MS/MS assay development in a clinical laboratory for the routine quantification of steroids and other biomarkers in human biofluids. QC materials of commercially available TDM kits proved to be an effective tool for this purpose.

## Data Availability

Raw data of the data presented in this study are available on request from the corresponding author. The data are not publicly available due to logistic reasons.

## Supplementary Materials

The following are available online at https://www.mdpi.com/article/10.3390/cells12020329/s1, Scheme S1: Steroid analytes and the respective interferences presented in this work. Chemical structures, sum formula, molecular weight and CAS number are depicted together with the substance name, and the abbreviation is used in parenthesis; Figure S1: Product ion spectra from precursor ion m/z 331 at CE 31V recorded by infusing a pure solution of PX; Figure S2: Influence of column LOT and injection matrix on RT of PX in comparison to 17P. (A) Overlay of ion transition chromatograms of PX, PX-IS, 17P and 17P-IS from injecting single-compound solutions in 50% methanol on three different columns (#6, #7 and #8). (B) Overlay of PX and 17P ion transition chromatograms from injecting a PBS solution of each compound that was processed by general sample preparation on the same three columns; Figure S3: Isotope pattern as calculated with the online tool “mstool” and chemical structure of alpha-hydroxytriazolam; Table S1: TDM panels used for interference checks.
