# Supplementary material for: Non-Steroidal Drug Interferences in a Quantitative Multisteroid LC-MS/MS Assay
Source: Cells. 2023 Jan 15;12(2):329. doi: 10.3390/cells12020329 (PMC9856384; doi:10.3390/cells12020329)
Supplement: Supplementary file 1 [file cells-12-00329-s001.zip › cells-2141959-supplementary.pdf]

Supplement

Non-steroidal Drug Interferences in a Quantitative Multisteroid LC-MS/MS Assay

Valentin Braun [1,2], Hermann Stuppner [1], and Christoph Seger [1,2,*]

[1] Institute of Pharmacy/Pharmacognosy, CCB—Centrum of Chemistry and Biomedicine, University of Innsbruck, Innrain 80-82, A-6020 Innsbruck, Austria
[2] Risch Ostschweiz AG, Lagerstrasse 30, 9470 Buchs, Switzerland
* Correspondence: christoph.seger@uibk.ac.at

Description of supporting material:

Scheme S1: Steroid analytes and the respective interferences presented in this work. Chemical structures, sum formula, molecular weight and CAS number is depicted together with the substance name and the abbreviation used in parenthesis.

Figure S1: Product ion spectra from precursor ion m/z 331 at CE 31V recorded by infusing a pure solution of PX.

Figure S2: Influence of column LOT and injection matrix on RT of PX in comparison to 17P. (A) Overlay of ion transition chromatograms of PX, PX-IS, 17P and 17P-IS from injecting single-compound solutions in 50% methanol on three different columns (#6, #7, #8). (B) Overlay of PX and 17P ion transition chromatograms from injecting a PBS solution of each compound that was processed by general sample preparation on the same three columns.

Figure S3: Isotope pattern as calculated with the online tool "mstool" and chemical structure of alpha-hydroxytriazolam

Table S1: TDM panels used for interference checks.

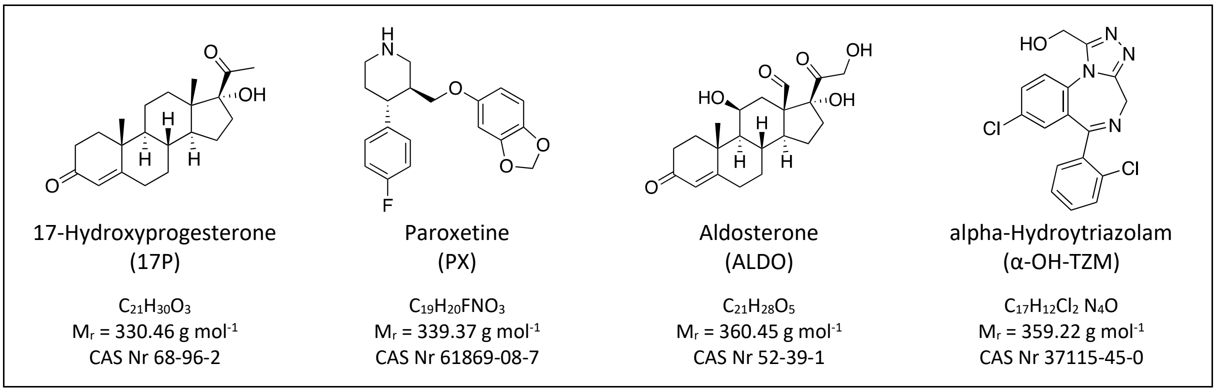

Scheme S1: Steroid analytes and the respective interferences presented in this work. Chemical structures, sum formula, molecular weight and CAS number is depicted together with the substance name and the abbreviation used in parenthesis.

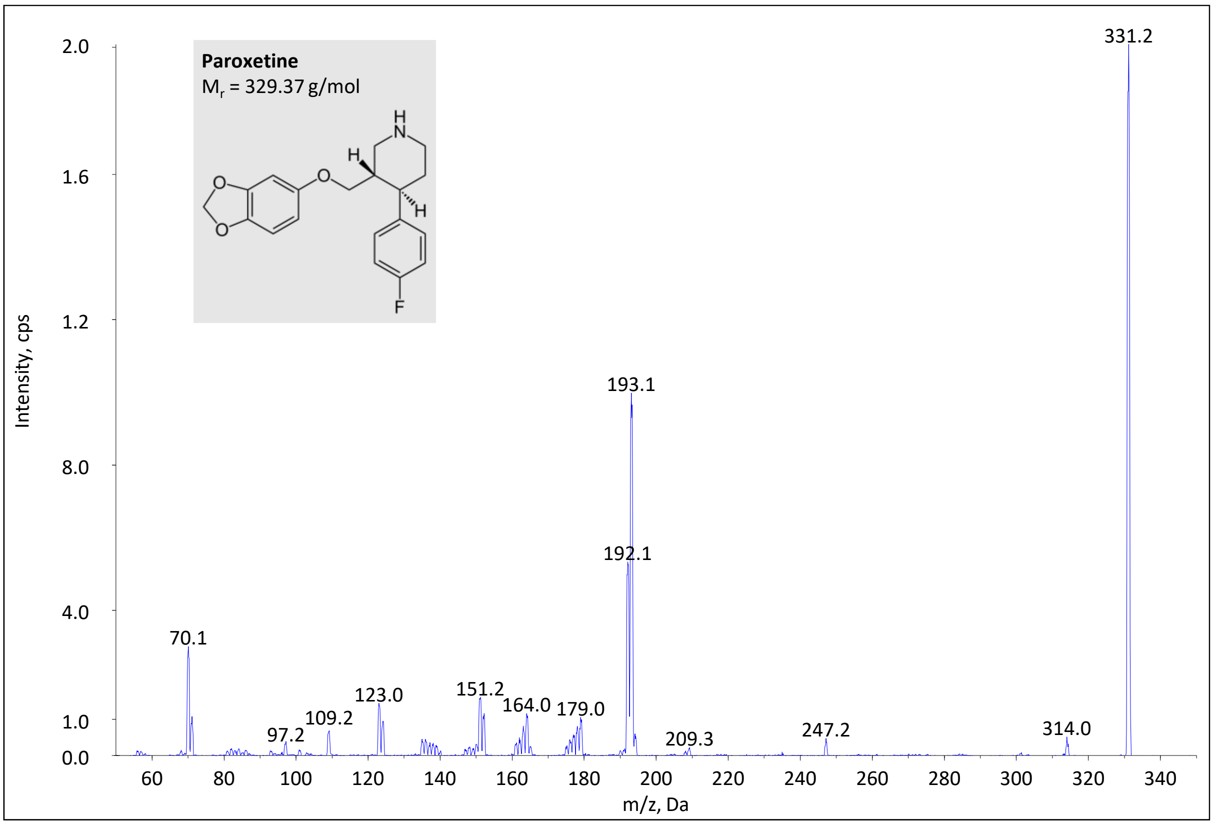

Figure S1: Product ion spectra from precursor ion m/z 331 at CE 31V recorded by infusing a pure solution of PX.

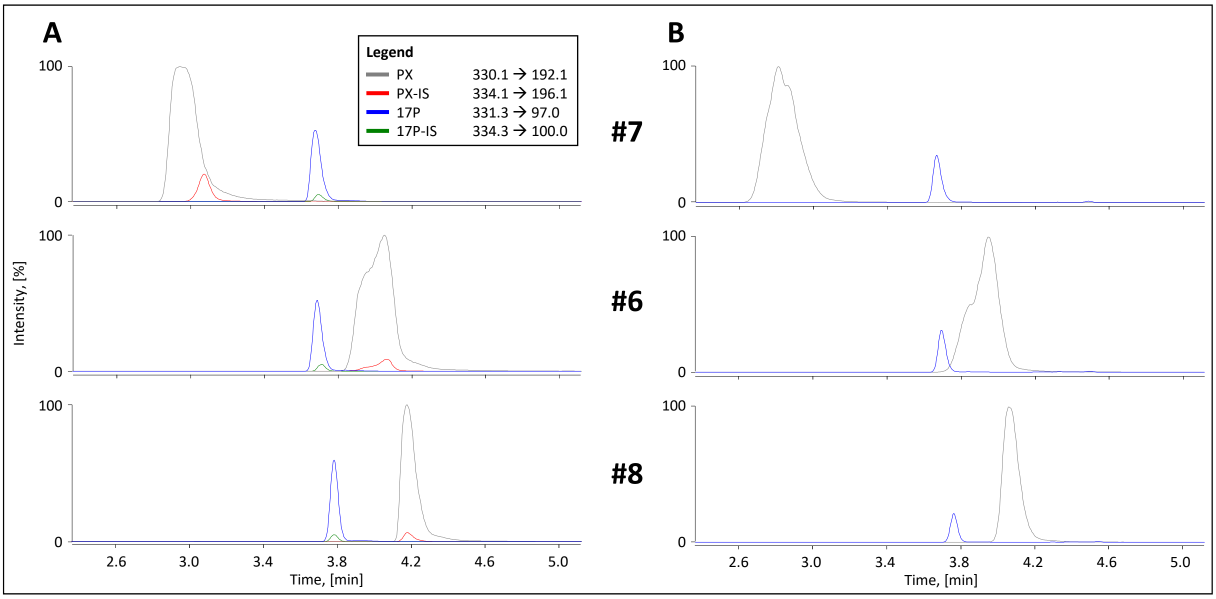

Figure S2: Influence of column LOT and injection matrix on RT of PX in comparison to 17P. (A) Overlay of ion transition chromatograms of PX, PX-IS, 17P and 17P-IS from injecting single-compound solutions in 50% methanol on three different columns (#6, #7, #8). (B) Overlay of PX and 17P ion transition chromatograms from injecting a PBS solution of each compound that was processed by general sample preparation on the same three columns.

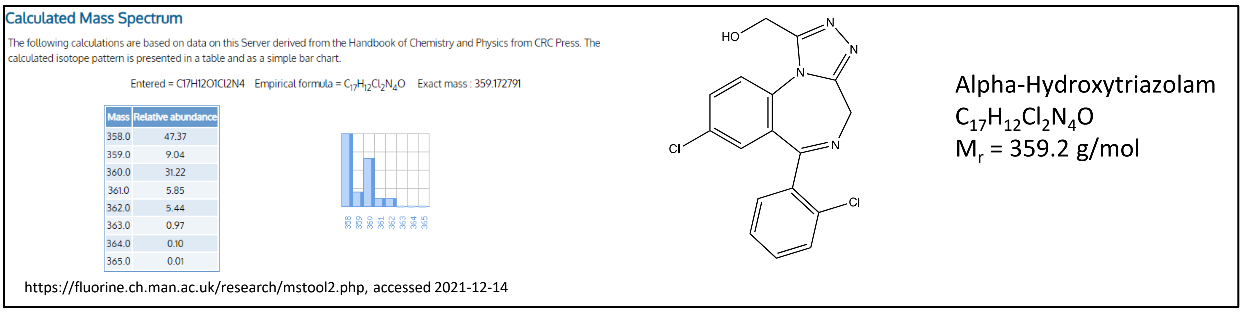

Figure S3: Isotope pattern as calculated with the online tool "mstool" and chemical structure of alpha-hydroxytriazolam

Table S1: Commerical TDM panels used for interference checks.

TDM panel name	Included Drugs and their molecular masses (g/mol)			
"Benzodiazepines panel 1"[1]	Chlordiazepoxide	299.8	Midazolam	325.8
	Clobazam	300.7	1-OH-Midazolam	341.8
	Norclobazam	286.7	Oxazepam	286.7
	Demoxepam	286.7	Prazepam	324.8
	Diazepam	284.7	Temazepam	300.7
	Nordiazepam	270.7	Tetrazepam	288.8
	Medazepam	270.8		
"Benzodiazepines Panel 2"[1]	Alprazolam	308.8	Desalkylflurazepam	288.7
	Bromazepam	316.2	Lorazepam	321.2
	Clonazepam	315.7	Lormetazepam	335.2
	Flunitrazepam	313.3	Nitrazepam	281.3
	Flurazepam	387.9	Triazolam	343.2
"Neuroleptics Panel 1"[1]	Aripiprazol	448.4	Olanzapine	312.4
	Dehydroaripiprazol	446.4	Quetiapine	383.5
	Clozapine	326.8	Norquetiapine	295.4
	N-Desmethylclozapine	312.8	Risperidone	410.5
	Haloperidol	375.9	9-OH-Risperidone	426.5
	N-Desmethylolanzapine	298.4		
"Neuroleptics Panel 2"[1]	Amisulpride	369.5	Melperone	263.4
	Benperidol	381.4	Perazine	339.5
	Brexipiprazole	433.6	Perphenazine	404.0
	Bromperidol	420.3	Pimozide	461.6
	Cariprazine	427.4	Pipamperone	375.5
	Chlorpromazine	318.9	Promethazine	274.4
	Chlorprothixene	315.9	Prothipendyl	285.4
	Flupentixol	434.5	Sertindole	440.9
	Fluphenazine	437.5	Sulforidazine	402.6
	Guanfacine	246.1	Sulpiride	341.4
	Ilperidone	426.5	Thioridazine	370.6
	Levomepromazine	328.5	Ziprasidone	412.9
	Loxapine	327.8	Zotepine	331.9
	Lurasidone	492.7	Zuclopenthixol	401.0
"Tricyclic Antidepressants Panel 1"[1]	Amitryptiline	277.4	Desipramine	266.4
	Doxepin	279.4	Nortriptyline	263.4
	Nordoxepin	265.4	Imipramine	280.4
"Tricyclic Antidepressants Panel 2"[1]	Clomipramine	314.9	Protriptyline	263.4
	Norclomipramine	300.8	Trimipramine	294.4
	Maprotiline	277.4	Nortrimipramine	280.4
	Normaprotiline	263.4		
"Antidepressants Panel 1"[1]	Citalopram	324.4	N-Desmethylmirtazapine	251.3
	N-Desmethylcitalopram	310.4	Paroxetine	329.4
	Duloxetine	297.4	Sertraline	306.2
	Fluoxetine	309.3	N-Desmethylsertraline	292.2
	Desmethylfluoxetine	295.3	Venlafaxine	277.4
	Fluvoxamine	318.3	O-Desmethylvenlafaxine	263.4
	Mirtazapine	265.4		
"Antidepressants Panel 2"[1]	Atomoxetine	255.4	Milnacipran	246.4
	Bupropion	239.7	Moclobemide	268.7
	Erythro-Dihydrobupropion	241.8	Opipramol	363.5
	Hydroxybupropion	255.7	Reboxetine	313.4
	Threo-Dihydrobupropion	241.8	Ritalinic Acid	219.3

TDM panel name	Included Drugs and their molecular masses (g/mol)			
	Clomethiazole	161.7	Tianeptine	437.0
	Dosulepin	295.5	Tranylcypromine	133.2
	N-Desmethyldosulepin	281.4	Trazodone	371.9
	Methylphenidate	233.3	Vilazodone	441.5
	Mianserin	264.4	Vortioxetine	298.5
"Antiepileptic Drugs"[1]	Brivaracetam	212.3	Phenylethylmalonamide	206.2
	Carbamazepine	236.3	Phenytoin	252.3
	Carbamazepine-10,11-epoxide	252.3	Pregabalin	159.2
	10,11-Dihydroxycarbamazepine	270.3	Primidone	218.3
	10-OH-Carbamazepine	252.3	Retigabine	303.3
	Oxcarbamazepine	252.3	Rufinamide	238.2
	Ethosuximide	141.2	Stiripentol	234.3
	Felbamate	238.2	Sultiame	290.4
	Gabapentin	171.2	Theophylline	180.2
	Lacosamide	250.3	Tiagabine	375.6
	Lamotrigine	256.1	Topiramate	339.4
	Levetiracetam	170.2	Valproic acid	144.2
	N-Desmethylmesuximide	189.2	Vigabatrin	129.2
	Perampanel	349.4	Zonisamide	212.2
	Phenobarbital	232.2		
"Antiarrhymtic Drugs"[1]	Acebutolol	336.4	Gallopamil	484.6
	Ajmaline	326.4	Lidocaine	234.3
	Amiodarone	645.3	Metoprolol	267.4
	Desethylamiodarone	617.3	Mexiletine	179.3
	Aprindine	322.5	Propafenone	341.4
	Atenolol	266.3	Propranolol	259.3
	Bisoprolol	325.5	Quinidine	324.4
	Diltiazem	414.5	Hydroquinidine	326.4
	Disopyramide	339.5	Sotalol	272.4
	Dronedarone	556.8	Tocainide	192.3
	Debutyldronedarone	500.7	Verapamil	454.6
	Flecainide	414.3	Norverapamil	440.6
	Flunarizine	404.5		
"Antidepressant drugs"[2]	Ritalinic acid	219.3	Reboxetine	313.4
	O-Desmethyltramadol	249.4	Citalopram	324.4
	O-Desmethylvenlafaxine	263.4	Opipramol	363.5
	Moclobemide	268.7	Desmethylmianserin	250.3
	Guanfacine	246.1	Bupropion	239.7
	Tramadol	263.4	Paroxetine	329.4
	Hydroxybupropion	255.7	Atomoxetine	255.4
	Clomethiazole	161.7	Fluoxamine	318.3
	Methylphenidat	233.3	Duloxetine	297.4
	Tianeptin	437.0	Desmethylfluoxetine	295.3
	Milnacipran	246.4	Dosulepin	295.5
	Desmethylmirtazapin	251.3	Fluoxetine	309.3
	Agomelatine	243.3	Desmethylsertraline	292.2
	Venlafaxine	277.4	Mianserin	264.4
	Dihydro-Bupropion	241.5	Nefazodone	470.0
	Desmethylcitalopram	310.4	Sertralin	306.2
	Trazodone	371.9	Vortioxetin	298.5
"Benzodiazepines"[2]	7-Aminonitrazepam	251.3	Chlordiazepoxide	299.8
	7-Aminoclonazepam	285.7	Alprazolam	308.8
	3-Hydroxybromazepam	332.2	alpha-OH-Midazolam	341.8

TDM panel name	Included Drugs and their molecular masses (g/mol)			
	7-Aminoflunitrazepam	283.3	Triazolam	343.2
	Bromazepam	316.2	Desalkylflurazepam	288.7
	Demoxepam	286.7	Flunitrazepam	313.3
	Zopiclone	388.7	Temazepam	300.7
	Zaleplon	305.3	Brotizolam	393.7
	Alpha-OH-Triazolam	359.2	Nordiazepam	270.7
	Alpha-OH-Alprazolam	324.8	Clobazam	300.7
	Desmethylflunitrazepam	299.3	Lormetazepam	335.2
	Oxazepam	286.7	Trazodone	371.9
	Nitrazepam	281.3	Flurazepam	387.9
	Estazolam	294.7	Midazolam	325.8
	Norclobazam	286.7	Diazepam	284.7
	Lorazepam	321.2	Tetrazepam	288.8
	Clonazepam	315.7	Prazepam	324.8
	Zolpidem	307.4	Medazepam	270.8

[1]TDM Panels from Chromsystems®; [2]TDM Panels from Recipe®
